# Cytotoxic and Antibacterial Compounds from the Coral-Derived Fungus *Aspergillus tritici* SP2-8-1

**DOI:** 10.3390/md15110348

**Published:** 2017-11-07

**Authors:** Weiyi Wang, Yanyan Liao, Chao Tang, Xiaomei Huang, Zhuhua Luo, Jianming Chen, Peng Cai

**Affiliations:** 1 Key Laboratory of Urban Environment and Health, Institute of Urban Environment, Chinese Academy of Sciences, Xiamen 361021, China; wywang@iue.ac.cn (W.W.); yyliao@iue.ac.cn (Y.L.); ctang@iue.ac.cn (C.T.); xmhuang@iue.ac.cn (X.H.); 2 University of Chinese Academy of Sciences, Beijing 100049, China; 3 State Key Laboratory Breeding Base of Marine Genetic Resources, Key Laboratory of Marine Genetic Resources, Fujian Key Laboratory of Marine Genetic Resources, Fujian Collaborative Innovation Centre for Exploitation and Utilization of Marine Biological Resources, Third Institute of Oceanography, State Oceanic Administration, Xiamen 361005, China; luozh_fj@163.com; 4 Xiamen Key Laboratory of Physical Environment, Xiamen 361021, China; 5 Institute of Oceanography, Minjiang University, Fuzhou 350108, China

**Keywords:** *Aspergillus*, candidusin, aspetritone, cytotoxic, antibacterial

## Abstract

Three novel compounds, 4-methyl-candidusin A (**1**), aspetritone A (**2**) and aspetritone B (**3**), were obtained from the culture of a coral-derived fungus *Aspergillus tritici* SP2-8-1, together with fifteen known compounds (**4**–**18**). Their structures, including absolute configurations, were assigned based on NMR, MS, and time-dependent density functional theory (TD-DFT) ECD calculations. Compounds **2** and **5** exhibited better activities against methicillin-resistant strains of *S. aureus* (MRSA) ATCC 43300 and MRSA CGMCC 1.12409 than the positive control chloramphenicol. Compound **5** displayed stronger anti-MRSA and lower cytotoxic activities than **2**, and showed stronger antibacterial activities against strains of *Vibrio vulnificus*, *Vibrio rotiferianus*, and *Vibrio campbellii* than the other compounds. Compounds **2** and **10** exhibited significantly stronger cytotoxic activities against human cancer cell lines HeLa, A549, and Hep G2 than the other compounds. Preliminary structure–activity relationship studies indicated that prenylation of terphenyllin or candidusin and the tetrahydrobenzene moiety in anthraquinone derivatives may influence their bioactivity.

## 1. Introduction

To date, approximately 70,000 species of fungi have been characterized [1]. Among them, about 1500 species of marine-derived fungi were mentioned, mainly from coastal ecosystems [1]. In recent years, the fungal sources of novel metabolites have broadened from saprophytic terrestrial strains to marine habitats and living plants with their endophytes [2]. Specifically, metabolites isolated from species of the genus *Aspergillus* have continually attracted the interest of pharmacologists due to their broad array of biological activities and their structural diversity. *A. tritici*, *A. campestris*, *A. taichungensis*, and *A. candidus*, which are members of the *Aspergillus* section *Candidi*, are known to be the prolific producers of bioactive secondary metabolites, including terphenyllin, candidusins, and anthraquinones [3]. 

**Figure 1:**
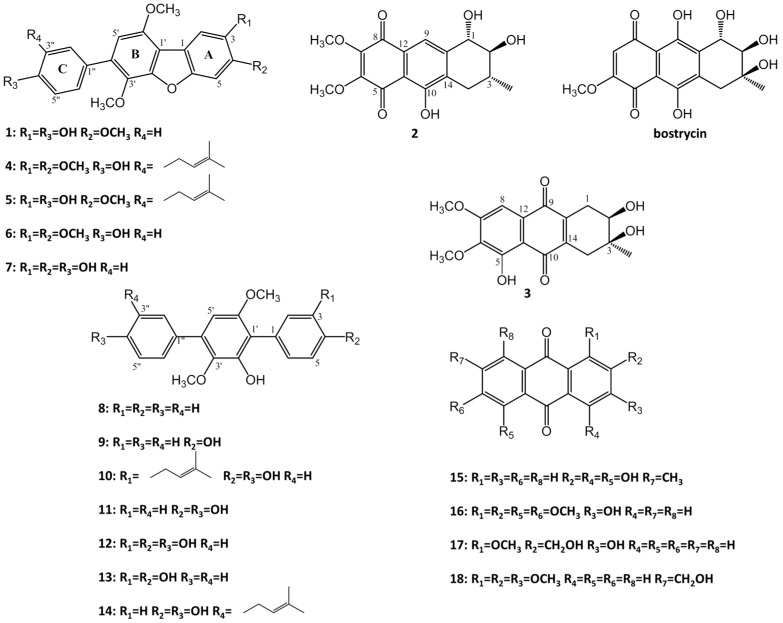
Structure of compounds **1**–**18**.

As part of our ongoing efforts to discover bioactive compounds from coral-derived microorganisms, an *Aspergillus tritici* strain, SP2-8-1, isolated from the coral of *Galaxea fascicularis*, collected at Port Dickson, Malaysia, attracted our attention. Studies on the bioactive constituents of its extract led to the isolation of three novel compounds, 4-methyl-candidusin A (**1**), aspetritone A (**2**) and aspetritone B (**3**), together with fifteen known analogues, including prenylcandidusin derivatives (**4–5**), candidusin derivatives (**6–7**), terphenyllin derivatives (**8–14**), and anthraquinone derivatives (**15–18**) (Figure 1).

## 2. Results and Discussion

**Figure 2:**
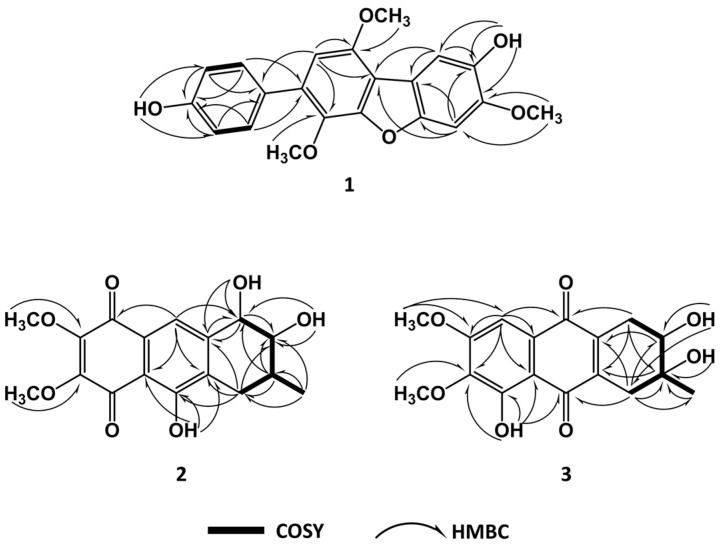
Key COSY and HMBC correlations of compounds **1**–**3**.

4-methyl-candidusin A (**1**) was obtained as a colorless amorphous solid. Its molecular formula was established as C_21_H_18_O_6_ by high-resolution electrospray ionization mass spectroscopy (HR-ESI-MS) (*m*/*z* 367.11757 [M + H]^+^; calcd for C_21_H_19_O_6_, 367.11816), implying 13 degrees of unsaturation. The ^13^C/distortionless enhancement by polarization transfer (DEPT) spectrum showed resonances for three methoxyl, seven methine, and 11 quaternary carbons. The ^1^H NMR spectrum displayed an AB system at *δ_H_* [6.85 (d, *J* = 8.53, H-3″, 5″) and 7.42 (d, *J* = 8.53, H-2″, 6″)]; three aromatic singlets at *δ_H_* [7.39 (s, H-2)], *δ_H_* [7.38 (s, H-5)], and *δ_H_* [6.72 (s, H-5′)]; three methoxyl singlets at *δ_H_* [3.87 (s, OCH_3_-4)], *δ_H_* [3.77 (s, OCH_3_-3′)], and *δ_H_* [3.97 (s, OCH_3_-6′)]; and two phenolic OH groups at *δ_H_* [9.06 (brs, OH-3)] and *δ_H_* [9.55 (brs, OH-4″)] (Table 1). In comparison with the previously reported candidusin A [4], the lack of a phenolic OH unit and the appearance of a methoxyl group in **1** were observed, confirmed by evidence of a 14 amu increase in the molecular weight of **1**. In addition, they shared the same substructures of rings B and C, with the main differences located on ring A. In combining the correlations of ^1^H–^1^H COSY and heteronuclear multiple bond correlation (HMBC) spectra (Figure 2) with the “no splitting” of H-2 and H-5, we assigned the structure of compound **1** as 4-methyl-candidusin A (**1**).

Aspetritone A (**2**) was obtained as a yellow amorphous solid. Its molecular formula was established as C_17_H_18_O_7_ by HRESIMS (*m*/*z* 333.0966 [M − H]^−^; calcd. for C_17_H_17_O_7_, 333.0974), implying nine degrees of unsaturation. The ^13^C NMR spectrum showed resonances for two methoxyl, one methyl, one methylene, four methine, and nine quaternary carbons. The ^1^H NMR spectrum displayed an aromatic proton *δ_H_* [7.65 (s, H-9)], and two methoxyls at *δ_H_* [3.99 (s, OCH_3_-6)] and *δ_H_* [3.95 (s, OCH_3_-7)]. In comparison with the published data on bostrycin [5,6], both the ^1^H NMR and ^13^C NMR were similar, suggesting that compound **2** was a bostrycin derivative. Analysis of 1D NMR, ^1^H–^1^H COSY, heteronuclear single quantum correlation (HSQC), and HMBC data revealed the presence of one 1,2-dihydroxy-3-methylbutane unit and one pentasubstituted naphthoquinone moiety. In the HMBC spectrum, correlations of H-1 with C-9 and C-13, and of H-4 with C-10 and C-14, indicated 1,2-dihydroxy-3-methylbutane was connected to the naphthoquinone by linkage of C-1 with C-13 and of C-4 with C-14 (Figure 2). The phenolic OH was attached to C-10 by HMBC correlations of *δ_H_* [12.18 (s, OH-10)] with C-10, C-11, and C-14. The aromatic proton *δ_H_* [7.65 (s, H-9)] showed HMBC correlations with C-1, C-8, C-11, and C-14, suggesting C-9 was unsubstituted and the two methoxy groups were attached to C-6 and C-7. Therefore, the planar structure of compound **2** was identified as 3, 9-deoxy-7-methoxybostrycin and named aspetritone A (**2**).

**Figure 3:**
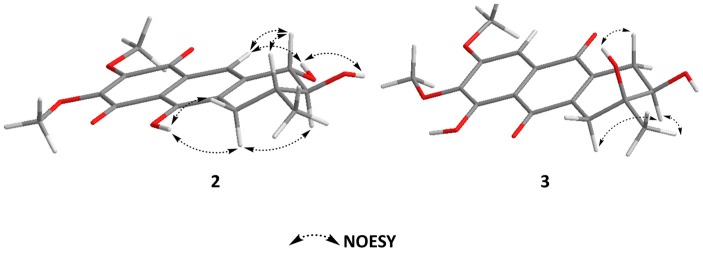
Key NOESY correlations of compounds **2** and **3**.

**Figure 4:**
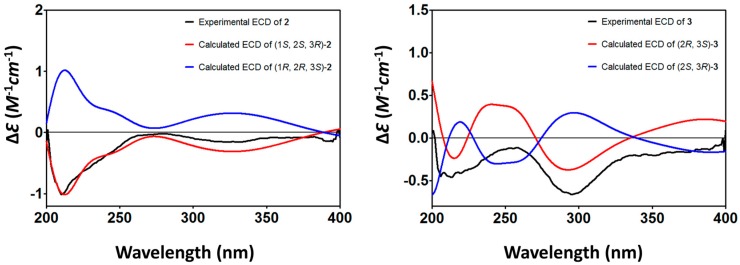
Calculated and Experimental ECD of compounds **2** and **3**.

The relative configuration of **2** was elucidated based on NOESY spectra (Figure 3). The strong NOESY correlations of H-1 with H-3 and of H-2 with H_ax_-4 indicated that both H-1 and H-3 faced to the same side of the tetrahydrobenzene ring and H-2 oriented to the opposite side. Therefore, two possible isomers of (1*S*, 2*S*, 3*R*)-**2** and (1*R*, 2*R*, 3S)-**2** were proposed, and their ECD spectra were calculated by time-dependent density functional theory (TD-DFT). The experimental ECD spectrum of **2** was in good agreement with the calculated ECD spectrum of (1*S*, 2*S*, 3*R*)-**2** (Figure 4), and the axial–axial coupling constants of ^3^*J*_Hax-1, H-2_ (7.03) and ^3^*J*_Hax-3, Hax-4_ (11.46) indicated a half-chair form of the tetrahydrobenzene ring with all of OH-1, OH-2, and CH_3_-3 in equatorial positions. In combining the NOESY correlations with the proton coupling constants, the absolute configuration of **2** was established as (1*S*, 2*S*, 3*R*)-3, 9-deoxy-7-methoxybostrycin (**2**).

Aspetritone B (**3**) was obtained as a yellow amorphous solid, and its molecular formula was determined as C_17_H_18_O_7_ by HRESIMS (*m*/*z* 333.0979 [M − H]^−^; calcd. for C_17_H_17_O_7_, 333.0974), implying nine degrees of unsaturation. The ^13^C NMR spectrum showed resonances for two methoxyl, one methyl, two methylene, two methine, and ten quaternary carbons. The ^1^H NMR spectrum displayed an aromatic proton *δ_H_* [7.16 (s, H-8)], and two methoxyls at *δ_H_* [3.79 (s, OCH_3_-6)] and *δ_H_* [3.92 (s, OCH_3_-7)]. In comparison with the published data of prisconnatanone A [7,8,9], both the ^1^H NMR and ^13^C NMR were similar, suggesting that compound **2** was a tetrahydroanthraquinone derivative. Analysis of 1D NMR, ^1^H-^1^H COSY, HSQC, and HMBC data revealed the presence of one 2,3-dihydroxy-3-methylbutane unit and one pentasubstituted naphthoquinone moiety. In HMBC spectra, correlations of H-1 with C-9 and C-14, and H-4 with C-10 and C-13, indicated that 2,3-dihydroxy-3-methylbutane was connected to the naphthoquinone by linkage of C-1 with C-13 and of C-4 with C-14. The phenolic OH was attached to C-5 by HMBC correlations of *δ_H_* [12.09 (s, OH-5)] with C-5, C-6, and C-11. The aromatic proton *δ_H_* [7.16 (s, H-8)] showed HMBC correlations with C-6, C-7, C-9, C-11, and C-12, suggesting C-8 was unsubstituted and the two methoxy groups were attached to C-6 and C-7 (Figure 2). Therefore, the planar structure of compound **3** was assigned as 1,2,3,4-tetrahydro-2,3,5-trihydroxy-3-methyl-6,7-dimethoxyanthracene-9,10-dione and named aspetritone B (**3**).

The relative configuration of **3** was elucidated based on NOESY spectra (Figure 3). The strong NOESY correlations of H_ax_-1 with OH-3 and of H-2 with CH_3_-3 indicated *cis*-configuration of OH-2 and OH-3. Therefore, two possible isomers of (2*R*, 3*S*)-**3** and (2*S*, 3*R*)-**3** were proposed, and their ECD spectra were calculated by TD-DFT. The experimental ECD spectrum of **3** was in good agreement with the calculated ECD spectrum of (2*R*, 3*S*)-**3** (Figure 4), and the axial–axial coupling constants of ^3^*J*_Hax-1, H-2_ (11.63) indicated a half-chair form of the tetrahydrobenzene ring with OH-2 and CH_3_-3 in equatorial positions. In combining the NOESY correlations with the proton coupling constants, the absolute configuration of **3** was established as (2*R*, 3*S*)-1,2,3,4-tetrahydro-2,3,5-trihydroxy-3-methyl-6,7-dimethoxyanthracene-9,10-dione (**3**).

The known compounds (**4–18**) were identified as 3,4-dimethyl-3″-prenylcandidusin A (**4**) [3], 4-methyl-3″-prenylcandidusin A (**5**) [3], 3,4-dimethyl-candidusin A (**6**) [3], candidusin A (**7)** [3], 4,4′-deoxy-terphenyllin (**8**) [10,11], 4″-deoxyterphenyllin (**9**) [10,11], 3-prenylterphenyllin (**10**) [10,11], terphenyllin (**11**) [12], 3-hydroxyterphenyllin (**12**) [13], 3-hydroxy-4″-deoxyterphenyllin (**13**) [11,14], 3″-prenylterphenyllin (**14**) [15], emodin (**15**) [16], 3-hydroxy- 1,2,5,6-tetramethoxyanthracene-9,10-dione (**16**) [16], 3-hydroxy-2-hydroxymethyl-1-methoxyanthracene-9,10-dione (**17**) [16], and 1,2,3-trimethoxy-7-hydroxymethylanthracene-9,10-dione (**18**) [16] respectively, by comparing their spectroscopic data with those reported in the literature.

The cytotoxic and antimicrobial activity of compounds **1–18** was evaluated using cell lines of HeLa, A549, and Hep G2, and strains of methicillin-resistant *Staphylococcus aureus* (MRSA) (ATCC 43300, CGMCC 1.12409), *Vibrio vulnificus* MCCC E1758, *Vibrio rotiferianus* MCCC E385, and *Vibrio campbellii* MCCC E333 (Table 2). In comparison to the positive control chloramphenicol, for strains of MRSA, compounds **2** and **5** exhibited better antibacterial activities, and compounds **1**, **3**, **4**, **7**, **9–12**, and **14–16** showed weaker activities. Compound **5** displayed stronger anti-MRSA and lower cytotoxic activities than compound **2**. For strains of *Vibrio*, compound **5** showed stronger antibacterial activities than the other compounds, with MIC values ranging from 7–15 μg/mL. For cytotoxicity, compounds **2** and **10** showed significantly stronger activities than the other compounds with IC_50_ values below 5 μM.

## 3. Materials and Methods

### 3.1. General Experimental Procedures

1D NMR and 2D NMR spectra were recorded on a Bruker DRX-400 instrument. HRESIMS was carried out on Bruker Daltonics Apex ultra 7.0 T Fourier transform mass spectrometer with an electrospray ionization source (Apollo II, Bruker Daltonics, Bremen, Germany). Optical rotations were measured with a P-1020 digital polarimeter (JASCO Corporation, Tokyo, Japan). CD spectra were measured on a J-715 spectropolarimeter (JASCO Corporation). The UV spectra were recorded on a UV-1800 spectrophotometer (Shimadzu, Japan). Thin-layer chromatography (TLC) plates (5 × 10 cm) were performed on GF254 (Branch of Qingdao Marine Chemical Co. Ltd., Qingdao, China) plates. For column chromatography (CC), RP-C18 (ODS-A, 50 µm, YMC, Kyoto, Japan), silica gel (200–300 mesh, 300–400 mesh, Branch of Qingdao Marine Chemical Co. Ltd., Qingdao, China), and Sephadex LH-20 (GE Healthcare Bio-Science AB, Pittsburgh, PA, USA) were used. The high performance liquid chromatography (HPLC) analysis was performed on a Waters 2695–2998 system (Waters, Milford, CT, USA). Semi-preparative HPLC was run with a P3000 pump (CXTH, Beijing, China) and a UV3000 ultraviolet-visible detector (CXTH, Beijing, China), using a preparative RP-C18 column (5 µm, 20 × 250 mm, YMC, Kyoto, Japan).

### 3.2. Fungal Material

Strain SP2-8-1 of A. tritici was isolated from the coral Galaxea fascicularis collected at Port Dickson, Malaysia, and was identified by ITS sequence homology (100% similarity with A. tritici CBS 266.81 with Genbank Accession No. KP987088.1 (max score 972, e value 0.0, query cover 100%)). The fungal strain was inoculated into a 15 mL centrifuge tube containing 3 mL of potato dextrose medium and cultured at 28 °C at 150 rpm for 3 days. Total genomic DNA was extracted as described by Lai et al. [17]. The internal transcribed spacer (ITS) region of rDNA was amplified by PCR using primers ITS1 (5′-TCCGTAGGTGAACCTGCGG-3′) and ITS4 (5′-TCCTCCGCTTATTGATATGC-3′). The PCR mixture consisted of 12.5 μL Taq premix (TaKaRa, Beijing, China), 0.25 μL (10 μM) of each primer, 0.75 μL dimethyl sulfoxide (DMSO), 10.25 μL dd H_2_O, and 1 μL DNA template. After denaturation at 95 °C for 4 min, amplification was performed with 32 cycles of 30 s at 95 °C, 30 s at 55 °C, and 40 s at 72 °C, and a final extension at 72 °C for 7 min. The ITS1-5.8S-ITS2 rDNA sequence of the fungus has been submitted to GenBank with the accession number MF716581. A voucher specimen was deposited at the Third Institute of Oceanography, SOA, China. The working strain was prepared on potato dextrose agar slants and stored at 4 °C.

### 3.3. Fermentation, Extraction, and Isolation

Strain SP2-8-1 was cultured on PDA plates at 28 °C for 3 days. Then, six plugs (5 mm diameter) were transferred to 12 Erlenmeyer flasks (1 L), each containing 500 mL Czapek’s medium (sucrose 30 g/L, NaNO_3_ 3.0 g/L, MgSO_4_·7H_2_O 0.5 g/L, KH_2_PO4 1.0 g/L, FeSO_4_ 0.01 g/L and KCl 0.5 g/L) in sterile conditions. Erlenmeyer flasks were shaken on a rotary shaker at 28 °C and 120 rpm for 3 days to form seed cultures (1 × 10^8^ spores/mL). Next, seed cultures (40 × 100 mL) were transferred to flasks (40 × 1 L) containing 45 g of millet and 105 g of rice per flask. After 28 days, the fermented culture was dried, smashed, and extracted with ethyl acetate (EtOAc). The EtOAc extract (220 g) was partitioned between petroleum ether (PE) and H_2_O, and then between EtOAc and H_2_O. Removal of the solvent of the EtOAc extract gave 150 g of residue, which was subject to silica gel (200–300 mesh) column chromatography, eluting with PE-EtOAC (9:1, 8.5:1.5, 8:2, 7.5:2.5, 6:4, 5:5, 4:6, *V*:*V*) to yield seven fractions, A–G. Further separation of fraction B (8.5:1.5, 24 g) was applied to silica gel column chromatography using PE-acetone and semi-preparative HPLC (80% methanol in H_2_O, flow rate 12 mL/min) to give compounds **15** (16 mg), **18** (16 mg) and **16** (16 mg). Fraction C (8:2, 15 g) was further purified by semi-preparative HPLC (60% methanol in H_2_O, flow rate 8 mL/min) and Sephadex LH-20 (50% chloroform in methanol) to give compounds **17** (16 mg) and **2** (4.5 mg). Fraction D (7.5:2.5, 10 g) was further purified by silica gel (200–300 mesh) column chromatography, eluting with hexane-EtOAc (6:4, *V*:*V*) and Sephadex LH-20 (methanol) to obtain compounds **3** (3.8 mg), **1** (32 mg) and **5** (16.9 mg). Fraction E (6:4, 10 g) was further separated by semi-preparative HPLC (80% methanol in H_2_O, flow rate 8 mL/min), Sephadex LH-20 (85% methanol in H_2_O), and preparative TLC to obtain compounds **4** (12 mg), **6** (15 mg), and **7** (20 mg). Fraction F (5:5, 20 g) was further separated by semi-preparative HPLC (80% methanol in H_2_O, flow rate 8 mL/min) and Sephadex LH-20 (methanol) to obtain compounds **8** (24 mg), **9** (15 mg), and **12** (25 mg). Fraction G (4:6, 32 g) was further separated by semi-preparative HPLC (80% methanol in H_2_O, flow rate 8 mL/min) and Sephadex LH-20 (methanol) to obtain compounds **11** (120 mg), **10** (60 mg), **13** (20 mg), and **14** (15 mg).

4-methyl-candidusin A (**1**): colorless amorphous solid; UV λ_max_ (methanol) nm (log *ε*): 295 (4.19); ^1^H NMR and ^13^C NMR data are shown in Table 1; HR-ESI-MS: *m*/*z* 367.11757 [M + H]^+^ (Calcd. for 367.11816, C_21_H_19_O_6_).

Aspetritone A (**2**): yellow amorphous solid; ${\left[\alpha \right]}_{D}^{20.0}-350\left(c\;0.15,\;\mathrm{MeOH}\right);$ UV λ_max_ (methanol) nm (log *ε*): 257 (3.58); ^1^H NMR and ^13^C NMR data are shown in Table 1; HR-ESI-MS: *m*/*z* 333.0966 [M − H]^−^ (Calcd. for 333.0974, C_17_H_17_O_7_).

Aspetritone B (**3**): yellow amorphous solid; ${\left[\alpha \right]}_{D}^{20.0}-156\;\left(c\;0.6,\;\mathrm{MeOH}\right);$ UV λ_max_ (methanol) nm (log *ε*): 265 (3.73); 283 (3.53); ^1^H NMR and ^13^C NMR data are shown in Table 1; HR-ESI-MS: *m*/*z* 333.0979 [M − H]^−^ (Calcd. for 333.0974, C_17_H_17_O_7_).

### 3.4. Antibacterial Assay

Antibacterial activities against MRSA (ATCC 43300, CGMCC 1.12409), *V. rotiferianus* (MCCC E385), *V. vulnificus* (MCCC E1758), and *V. campbellii* (MCCC E333) were tested by continuous dilution in 96-well plates using resazurin as a surrogate indicator. Blue resazurin was reduced by metabolically active bacteria to pink resorufin. A mid-logarithmic-phase tested strain was added at a starting inoculum of 5 × 10^5^ CFU/mL to the plate containing tested compound (final concentration ranging from 250 to 0.98 μg/mL in two-fold dilution) plus 10% resazurin solution (6.75 mg/mL in sterile water). The foil covered plate was incubated for 24 h with shaking at 37 °C. After that, by observing the blue-to-pink color change, the MIC value was determined to be the lowest concentration that did not induce the color change [18,19,20].

### 3.5. Cytotoxicity Assay

Hela (cervical cancer cell), Hep G2 (human liver cancer cell), and A549 (adenocarcinomic human alveolar basal epithelial cell) cells were maintained in DMEM, MEM, and F-12K medium respectively, and supplied with 10% FBS, 100 U/mL of penicillin, and 100 mg/mL of streptomycin [21]. Cells were grown in a humidified chamber with 5% CO_2_ at 37 °C. For cytotoxicity assays, cells were seeded at a density of 5000 cells per well in 96-well plates, grown at 37 °C for 12 h, and then treated with tested compound at five different concentrations (100 μL medium/well). The cytotoxicity was measured by Cell Counting Kit-8 (CCK-8) (DOJINDO) at 48 h post-treatment, following the manufacturer’s instructions. 

CCK-8 assay is based on the conversion of a tetrazolium salt, 2-(2-methoxy-4-nitrophenyl)-3-(4-nitrophenyl)-5-(2,4-disulfophenyl)-2*H*-tetrazolium, monosodium salt (WST-8), and a water-soluble formazan dye, upon reduction by dehydrogenases in the presence of an electronmediator [22]. WST-8 is reduced by dehydrogenases in cells to give an orange colored product (formazan). The amount of the formazan dye is directly proportional to the number of living cells.

In brief, 10 μL of CCK-8 solution was added to each well of the 96-well plates. After incubation at 37 °C for 2 h, the absorbance at 450 nm was measured using a SpectraMAX M5 microplate reader. Wells with only culture medium and CCK-8 solution were used to determine the background, and cells treated with DMSO were included as the negative controls [21].

### 3.6. ECD Calculation

Conformational analysis was initially performed using Confab [23] with the MMFF94 force field for all configurations. Room-temperature equilibrium populations were calculated according to the Boltzmann distribution law Equation (1). The conformers with Boltzmann-populations of over 1% were chosen for ECD calculations. The energies and populations of all dominative conformers were provided in Table S1.

(1) $$\frac{N_{i}}{N}=\frac{g_{i}e^{-\frac{E_{i}}{k_{B}T}}}{\sum g_{i}e^{-\frac{E_{i}}{k_{B}T}}}$$

*N_i_* is the number of conformer *i* with energy *E_i_* and degeneracy *g_i_* at temperature *T*, and *k*_B_ is the Boltzmann constant.

The theoretical calculation was carried out using Gaussian 09. First, the chosen conformer was optimized at PM6 using the semi-empirical theory method, and then optimized at B3LYP/6-311G** in methanol using the conductor-like polarizable continuum model (CPCM) (Table S2). The theoretical calculation of ECD was conducted in methanol using TD-DFT at the same theory level. Rotatory strengths for a total of 50 excited states were calculated. The ECD spectrum is simulated in SpecDis [24] by overlapping Gaussian functions for each transition.

## 4. Conclusions

In current research, we have isolated three novel compounds, 4-methyl candidusin A (**1**), aspetritone A (**2**), and aspetritone B (**3**), together with two prenylcandidusin derivatives (**4–5**), two candidusin derivatives (**6–7**), seven terphenyllin derivatives (**8–14**), and four anthraquinone derivatives (**15–18**). Candidusin can be deduced to be a cyclization product of terphenyllin between C-6 and C-2′ via an oxygen atom. C-prenylation plays an important role in diversification of natural compounds, especially for flavonoids and coumarins [25]. These compounds exhibit significant in vitro biological activities (cytotoxic, antibacterial, osteogenic, antioxidant, and anti-inflammatory activities) [25]. Prenylation in polyhydroxy-p-terphenyl analogues, as 3,4-dimethyl-3″-prenylcandidusin A (**4**), 4-methyl-3″-prenylcandidusin A (**5**), 3-prenylterphenyllin (**10**), and 3″-prenylterphenyllin (**14**), described in this paper, preliminarily influences the cytotoxicity and antibacterial activities, compared to the un-prenylated terphenyllin and candidusin derivatives. Chemical structures of compounds **2**, **3,** and **15–18** also preliminarily indicate that the special tetrahydrobenzene moiety located in compounds **2** and **3** attributed to their relatively strong bioactivity. Therefore, isolation or synthesis of more prenylated-terphenyllin, prenylated-candidusin, and tetrahydroanthraquinone derivatives deserves attention as an important aspect of structure–activity relationship studies.

## Figures and Tables

**Table 1:** ^1^H NMR data (400 MHz) and ^13^C NMR data (100 MHz) for compounds **1**–**3**.

Position	1		2		3	
	δ_H_, mult. (*J* in Hz)	δ_C_	δ_H_, mult. (*J* in Hz)	δ_C_	δ_H_, mult. (*J* in Hz)	δ_C_
1		114.9	4.26, d (7.03)	73.8	2.54, dd (19.84, 4.88), Heq 2.69, dd (19.84, 11.63), Hax	30.1
2	7.39, s	107.3	3.13, m	76.3	3.59, m	69.7
3		144.0	1.76, m	33.5		69.1
4		148.4	2.19, dd (18.45, 11.46), Hax 2.92, dd (18.45, 5.40), Heq	31.3	2.45, br d (13.30)	33.0
5	7.38, s	96.6		187.2		155.1
6		149.7		148.8		140.8
7				146.9		157.9
8				181.1	7.16, s	104.0
9			7.65, s	118.1		183.5
10				158.0		189.2
11				111.0		110.8
12				128.5		127.9
13				148.6		142.8
14				131.7		142.6
1′		114.2				
2′		149.0				
3′		136.4				
4′		131.4				
5′	6.72, s	106.0				
6′		150.0				
1″		129.0				
2″, 6″	7.42, d (8.53)	130.8				
3″, 5″	6.85, d (8.53)	115.5				
4″		157.2				
CH_3_-3			1.08, d (6.53)	18.5	1.21, s	26.0
OCH_3_-4	3.87, s	56.4				
OCH_3_-6			3.99, s	61.4	3.79, s	60.8
OCH_3_-7			3.95, s	61.7	3.92, s	56.8
OCH_3_-3′	3.77, s	61.0				
OCH_3_-6′	3.97, s	56.3				
OH-1			5.82, d (6.27)			
OH-2			5.06, d (5.02)		4.73, br s	
OH-3	9.06, brs				5.03, br s	
OH-5					12.09, s	
OH-10			12.18, s			
OH-4″	9.55, brs					

**Table 2:** Antibacterial and cytotoxic activities of compounds **1–18**. Data are expressed as mean ± SD values of three independent experiments, each made in triplicate.

Compound	MIC (μg/mL)					IC_50_ (μM)		
	MRSA 1	MRSA 2	VV	VR	VC	HeLa	A549	Hep G2
1	31.33 ± 0.61	30.97 ± 0.78	31.47 ± 1.22	NA	15.10 ± 0.44	30.23 ± 1.32	24.53 ± 1.10	27.50 ± 1.57
2	**7.53 ± 0.31**	**7.63 ± 0.21**	15.61 ± 0.48	31.17 ± 0.35	15.53 ± 0.60	**2.67 ± 0.60**	**3.13 ± 0.68**	**3.87 ± 0.74**
3	15.27 ± 0.35	15.63 ± 0.45	15.47 ± 0.51	31.33 ± 0.23	15.77 ± 0.29	10.57 ± 0.93	4.67 ± 0.60	8.57 ± 0.83
4	15.67 ± 0.50	7.57 ± 0.73	15.58 ± 0.33	15.57 ± 0.30	NA	16.77 ± 0.45	21.07 ± 0.76	27.17 ± 0.29
5	**3.80 ± 0.13**	**3.80 ± 0.22**	**7.77 ± 0.10**	**7.75 ± 0.18**	15.57 ± 0.30	10.20 ± 0.50	13.07 ± 0.72	35.10 ± 1.00
6	NA	NA	NA	NA	NA	NA	NA	NA
7	31.47 ± 0.24	31.23 ± 0.10	31.42 ± 0.23	31.33 ± 0.19	NA	25.07 ±0.81	19.07 ± 0.64	32.10 ± 2.00
8	NA	NA	NA	NA	NA	NA	NA	NA
9	31.30 ± 0.26	31.45 ± 0.22	31.37 ± 0.14	31.53 ± 0.31	31.47 ± 0.25	NA	NA	NA
10	15.53 ± 0.31	15.47 ± 0.23	31.43 ± 0.32	31.37 ± 0.21	NA	**3.23 ± 0.40**	**3.87 ± 0.15**	**2.10 ± 0.20**
11	31.47 ± 0.24	31.27 ± 0.16	31.37 ± 0.25	NA	NA	18.87 ± 1.27	12.33 ± 0.68	21.2 ± 0.35
12	31.30 ± 0.17	31.33 ± 0.12	31.43 ± 0.21	NA	NA	23.37 ± 0.84	36.07 ± 1.67	32.10 ± 2.65
13	NA	NA	NA	NA	NA	NA	NA	45.20 ± 1.00
14	31.33 ± 0.23	31.28 ± 0.10	31.25 ± 0.13	NA	31.43 ± 0.20	38.30 ± 1.50	NA	40.10 ± 0.90
15	15.65 ± 0.18	15.53 ± 0.12	15.73 ± 0.12	62.67 ± 0.15	31.35 ± 0.22	25.07 ± 0.81	22.17 ± 1.45	30.20 ± 0.87
16	31.32 ± 0.25	31.33 ± 0.23	NA	NA	NA	NA	NA	NA
17	NA	NA	NA	31.28 ± 0.14	NA	NA	45.63 ± 1.79	NA
18	NA	NA	NA	NA	NA	NA	NA	42.07 ± 1.07
erythromycin	NT	NT	1.92 ± 0.06	3.93 ± 0.03	7.68 ± 0.10	NT	NT	NT
chloramphenicol	7.67 ± 0.13	7.87 ± 0.08	NT	NT	NT	NT	NT	NT
doxorubicin	NT	NT	NT	NT	NT	0.50 ± 0.05	0.09 ± 0.01	1.06 ± 0.07

MRSA 1: methicillin-resistant *S. aureus* ATCC 43300; MRSA 2: methicillin-resistant *S. aureus* CGMCC 1.12409; VV: *V. vulnificus* MCCC E1758; VR: *V. rotiferianus* MCCC E385; VC: *V. campbellii* MCCC E333; NA: no activity at the concentration of 50 μg/mL (antibacterial) or 50 μM (cytotoxic); NT: not tested.

## Supplementary Materials

The following are available online at www.mdpi.com/1660-3397/15/11/348/s1: NMR spectra for compounds **1**–**3** as well as computational data for compounds **1**–**2**.
