# Supplementary material for: Cytotoxic and Antibacterial Compounds from the Coral-Derived Fungus Aspergillus tritici SP2-8-1
Source: Mar Drugs. 2017 Nov 7;15(11):348. doi: 10.3390/md15110348 (PMC5706038; doi:10.3390/md15110348)
Supplement: Supplementary file 1 [file marinedrugs-15-00348-s001.pdf]

marine drugs

Supplementary Files

Cytotoxic and Antibacterial Compounds from the Coral-Derived Fungus *Aspergillus tritici* SP2-8-1

Weiyi Wang [1,2,3,†], Yanyan Liao [1,2,4,†], Chao Tang [1,2,4], Xiaomei Huang [1,2,4], Zhuhua Luo [3], Jianming Chen [3,5,*], Peng Cai [1,2,4,*]

Content

Figure S1. ^{1}H NMR spectrum of compound 1
Figure S2. ^{13}C NMR spectrum of compound 1
Figure S3. ^{13}C/DEPT spectrum of compound 1
Figure S4. ^{1}H-^{1}H COSY spectrum of compound 1
Figure S5. HSQC spectrum of compound 1
Figure S6. HMBC spectrum of compound 1
Figure S7. HRESIMS spectrum of compound 1
Figure S8. UV spectrum of compound 1
Figure S9. ^{1}H NMR spectrum of compound 2
Figure S10. ^{13}C NMR spectrum of compound 2
Figure S11. ^{1}H-^{1}H COSY spectrum of compound 2
Figure S12. HSQC spectrum of compound 2
Figure S13. HMBC spectrum of compound 2
Figure S14. HRESIMS spectrum of compound 2
Figure S15. UV spectrum of compound 2
Figure S16. NOESY spectrum of compound 2
Figure S17. ^{1}H NMR spectrum of compound 3
Figure S18. ^{13}C NMR spectrum of compound 3
Figure S19. ^{1}H-^{1}H COSY spectrum of compound 3
Figure S20. HSQC spectrum of compound 3
Figure S21. HMBC spectrum of compound 3
Figure S22. HRESIMS spectrum of compound 3
Figure S23. UV spectrum of compound 3
Figure S24. NOESY spectrum of compound 3
Table S1. Energies of the dominative conformers at MMFF94 force field of compound 2 & 3
Table S2. Energies of the conformers at B3LYP/6-311G** of compound 2 & 3 in methanol
Text S1: ITS1-5.8S-ITS2 rDNA sequence of strain SP2-8-1

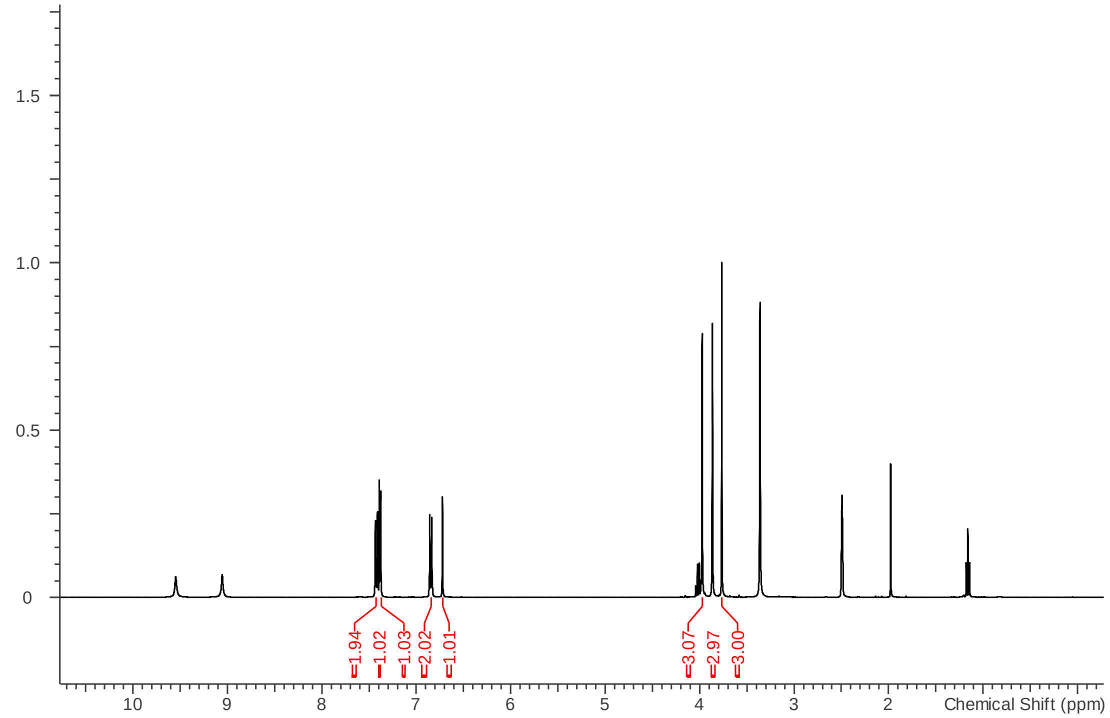

Figure S1. ^{1}H NMR spectrum of compound 1

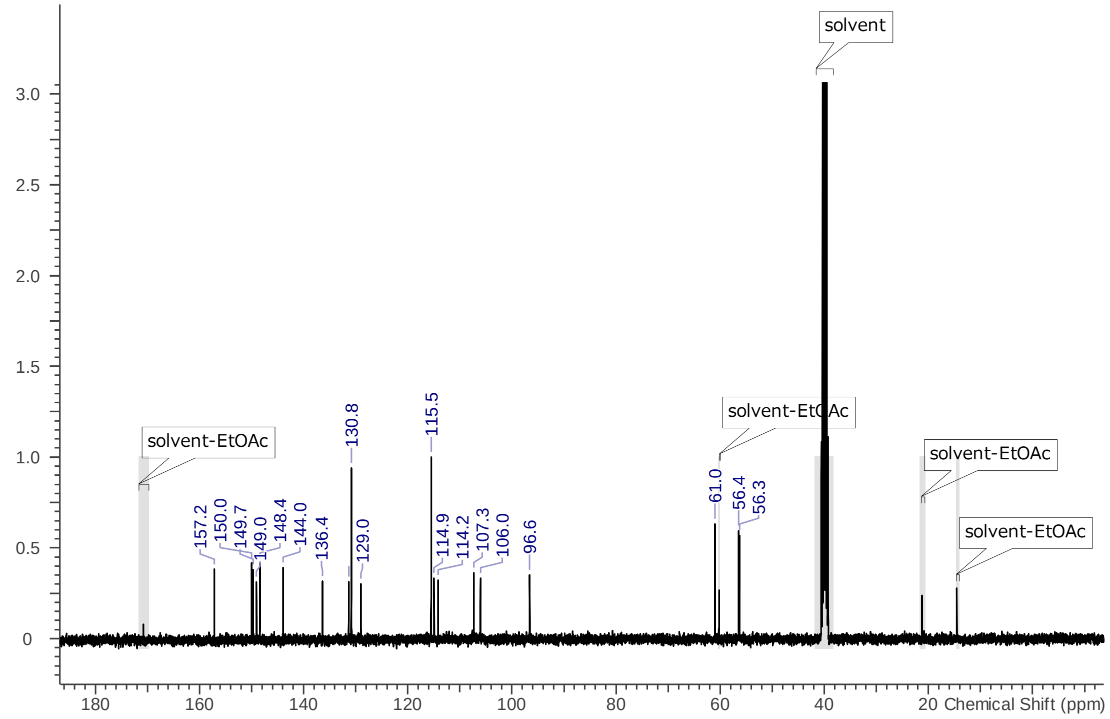

Figure S2. ^{13}C NMR spectrum of compound 1

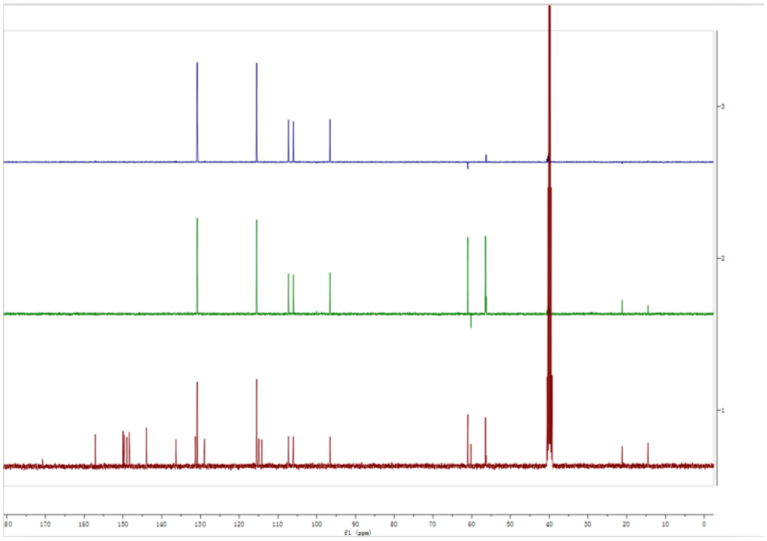

Figure S3. ^{13}C/DEPT spectrum of compound 1

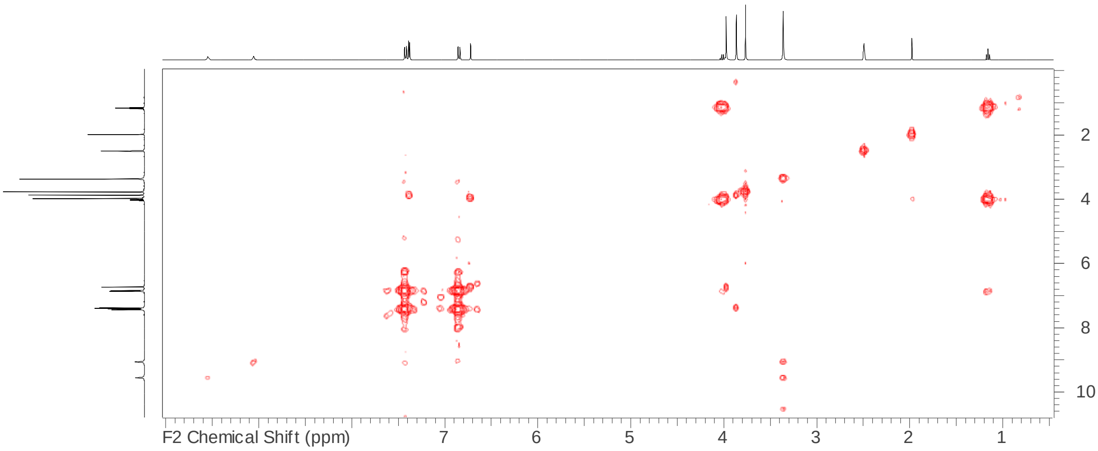

Figure S4. ^{1}H-^{1}H COSY spectrum of compound 1

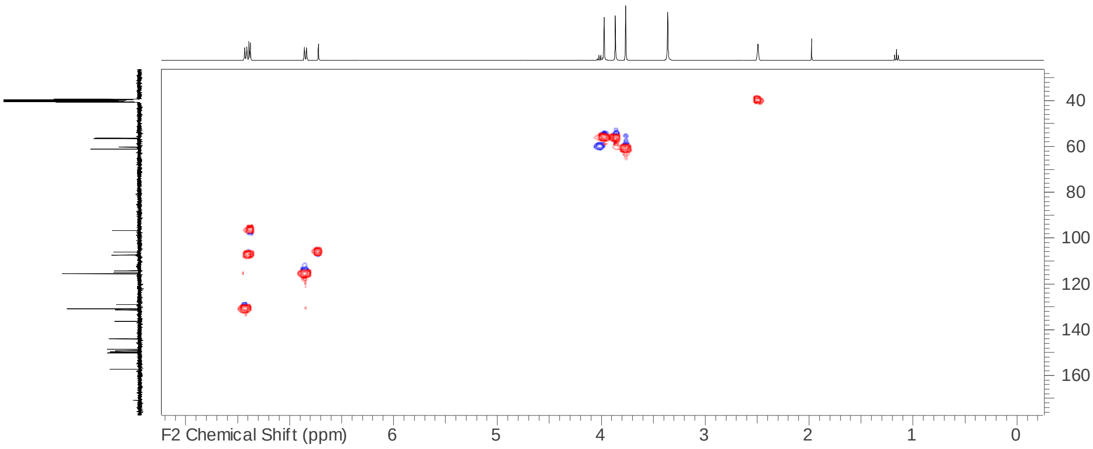

Figure S5. HSQC spectrum of compound 1

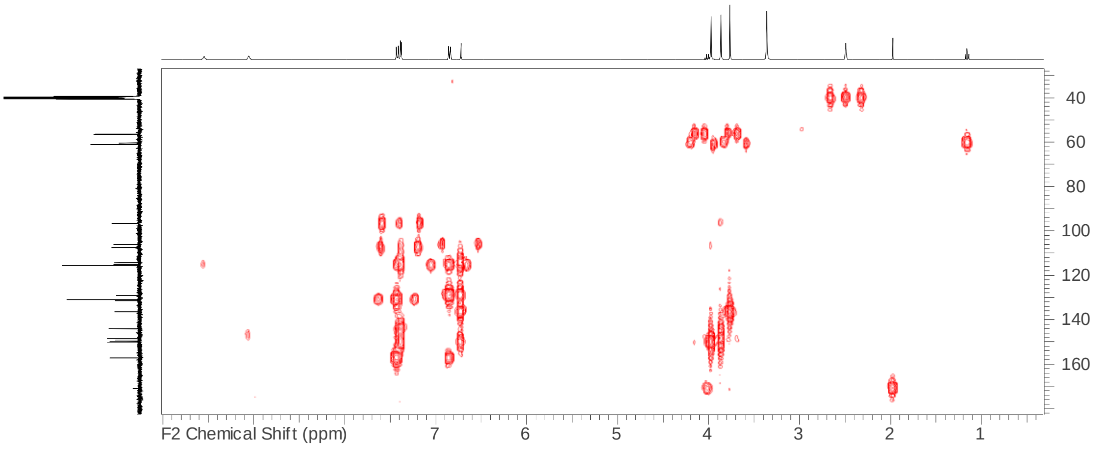

Figure S6. HMBC spectrum of compound 1

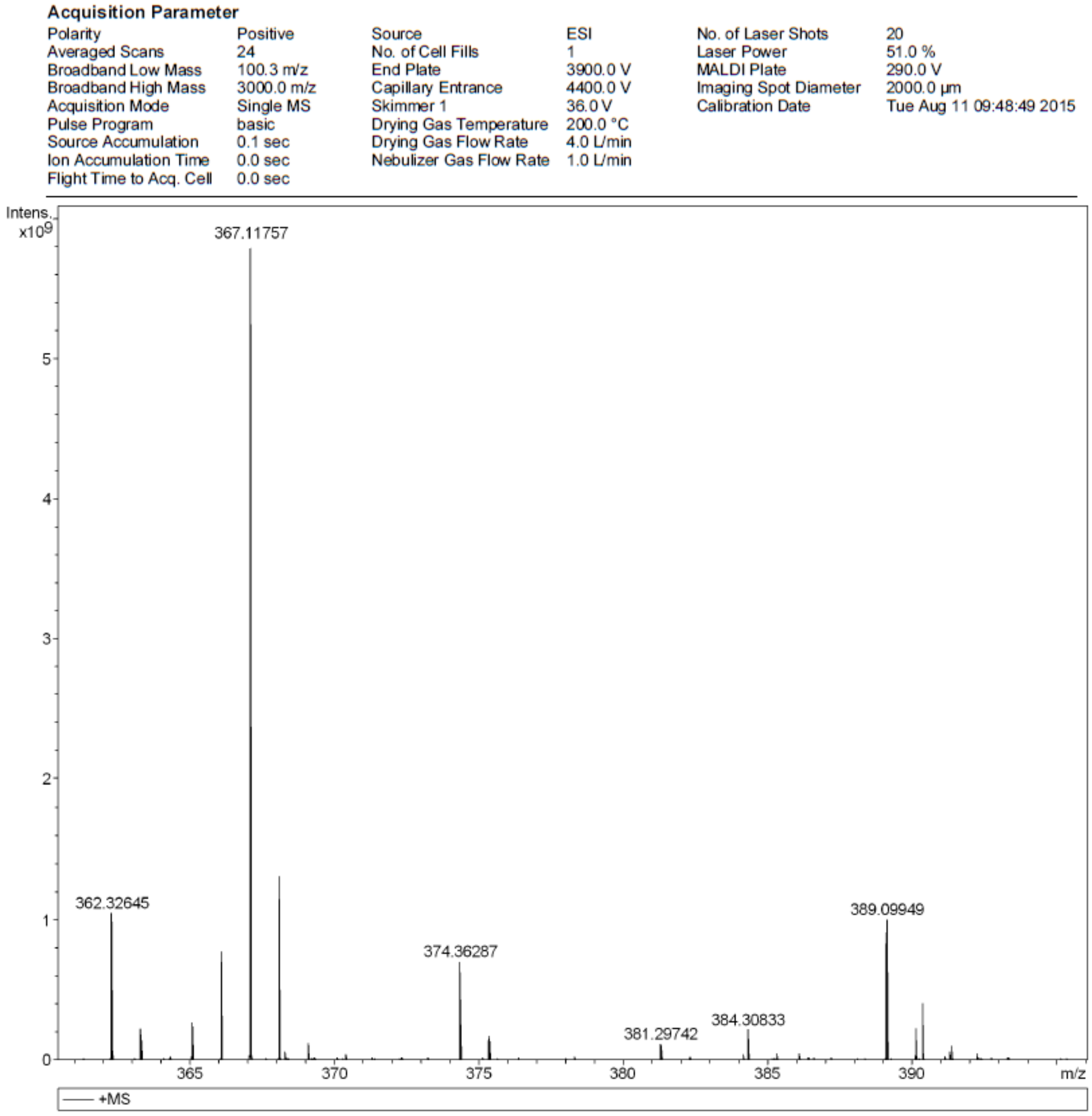

Figure S7. HRESIMS spectrum of compound 1

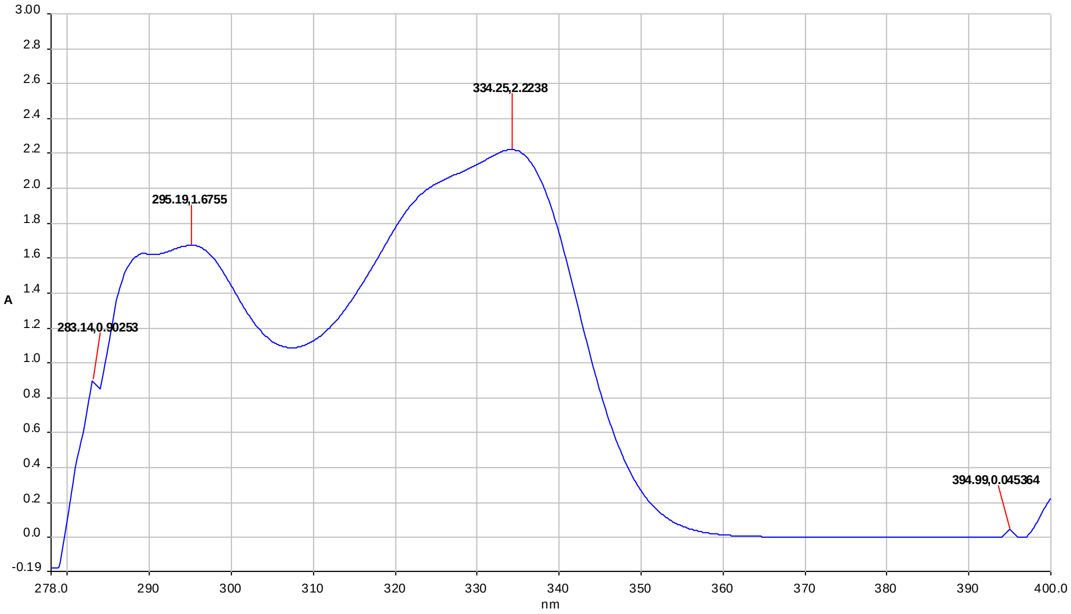

Figure S8. UV spectrum of compound 1

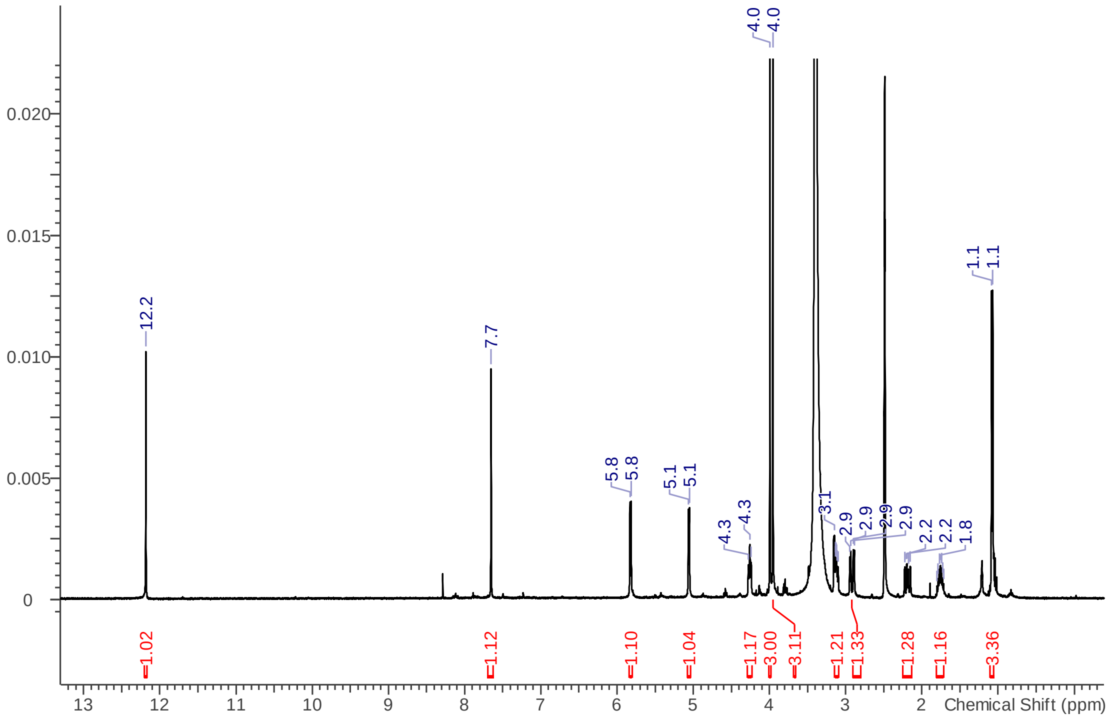

Figure S9. ^{1}H NMR spectrum of compound 2

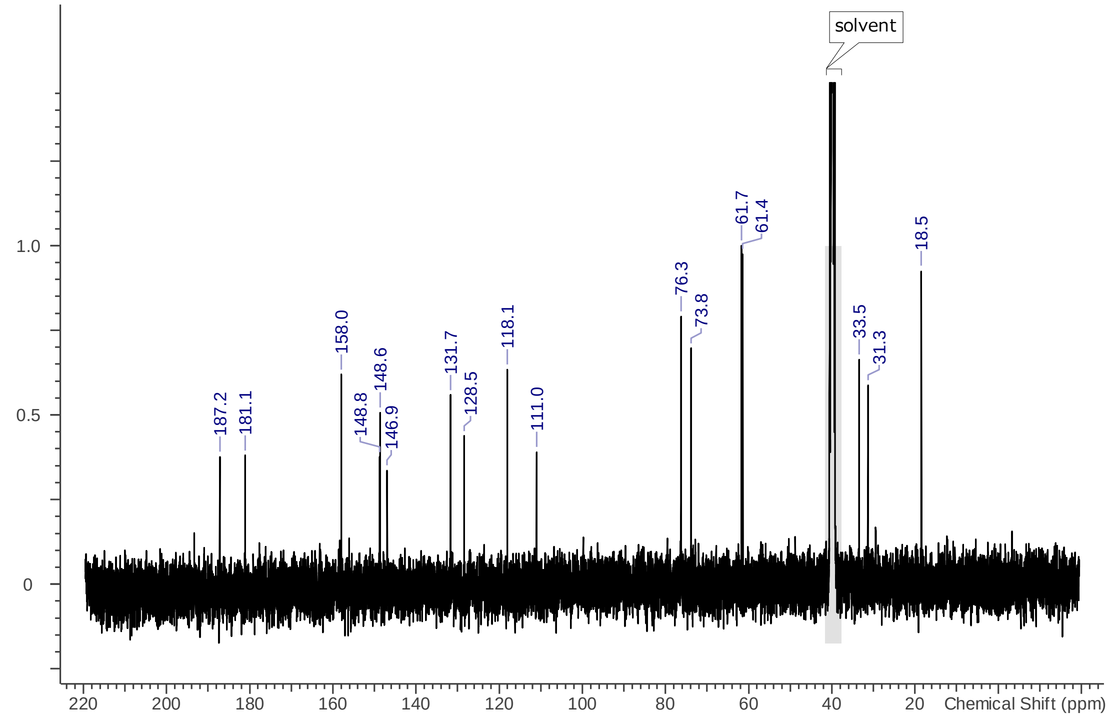

Figure S10. ^{13}C NMR spectrum of compound 2

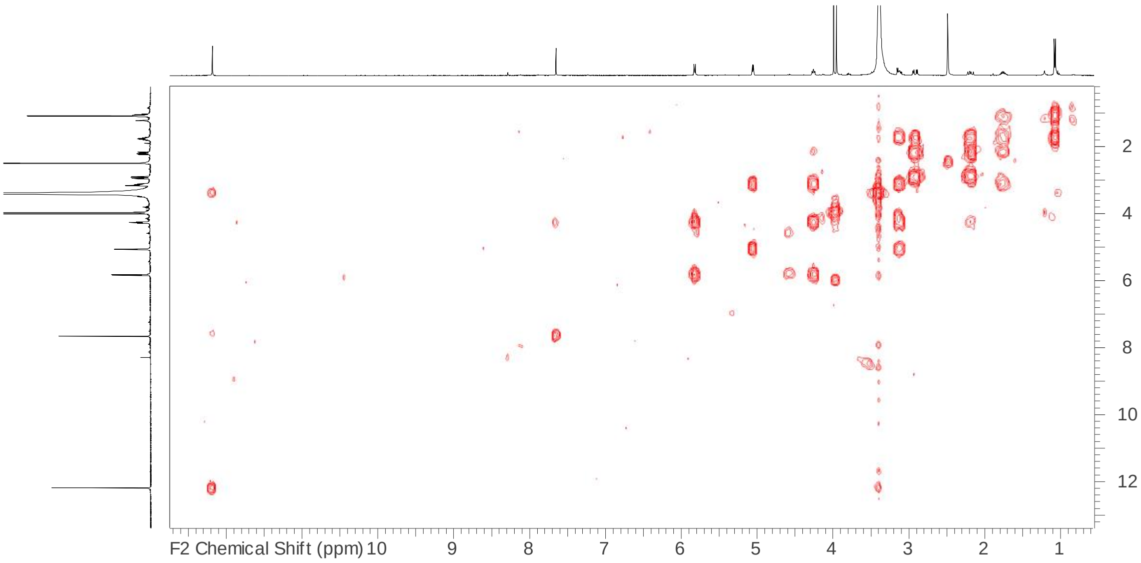

Figure S11. ^{1}H-^{1}H COSY spectrum of compound 2

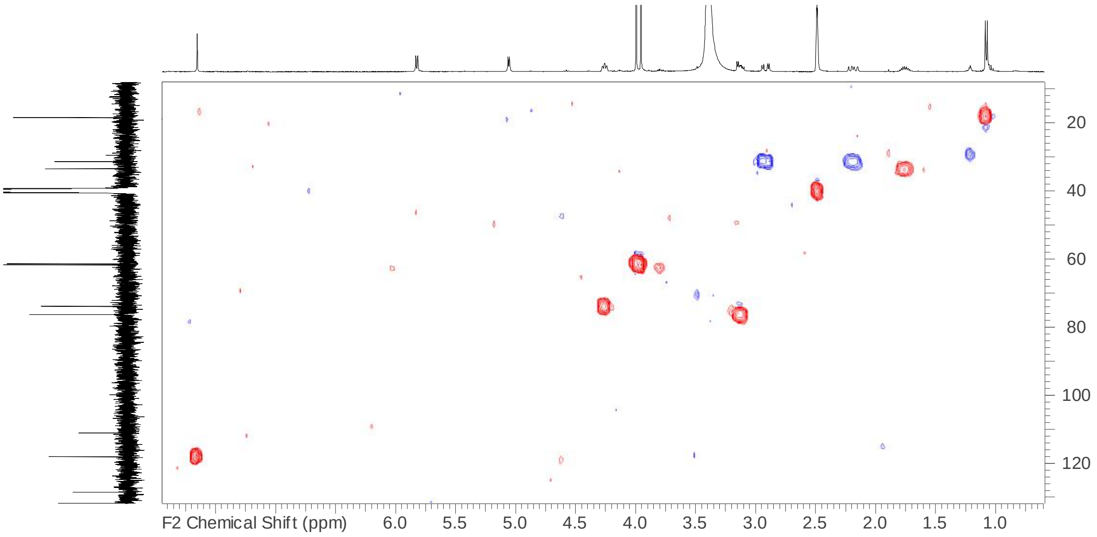

Figure S12. HSQC spectrum of compound 2

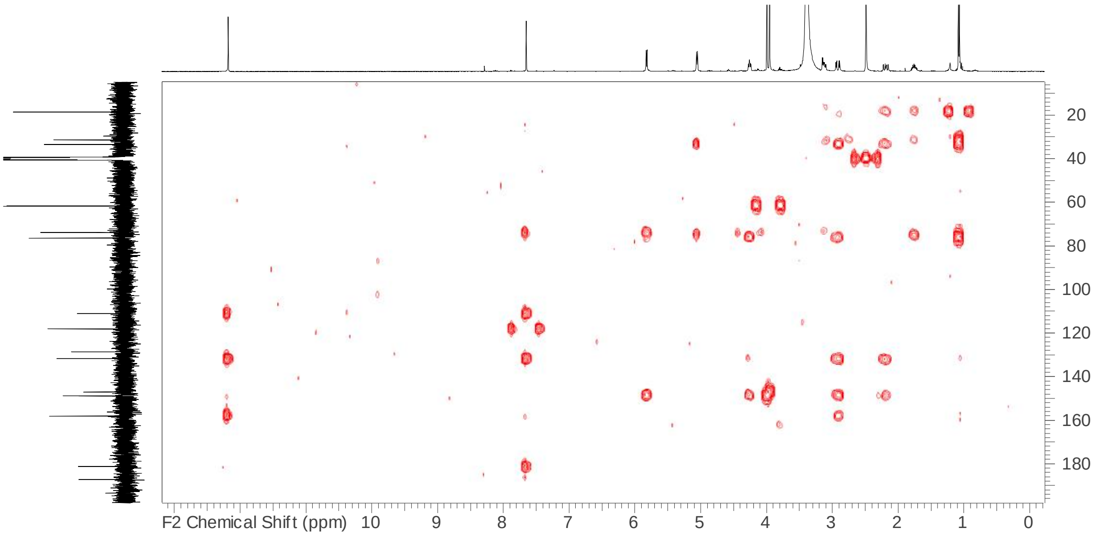

Figure S13. HMBC spectrum of compound 2

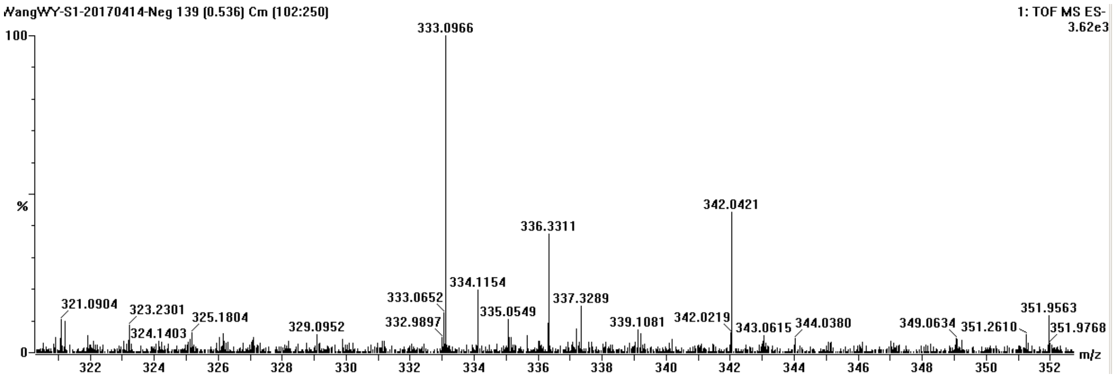

Figure S14. HRESIMS spectrum of compound 2

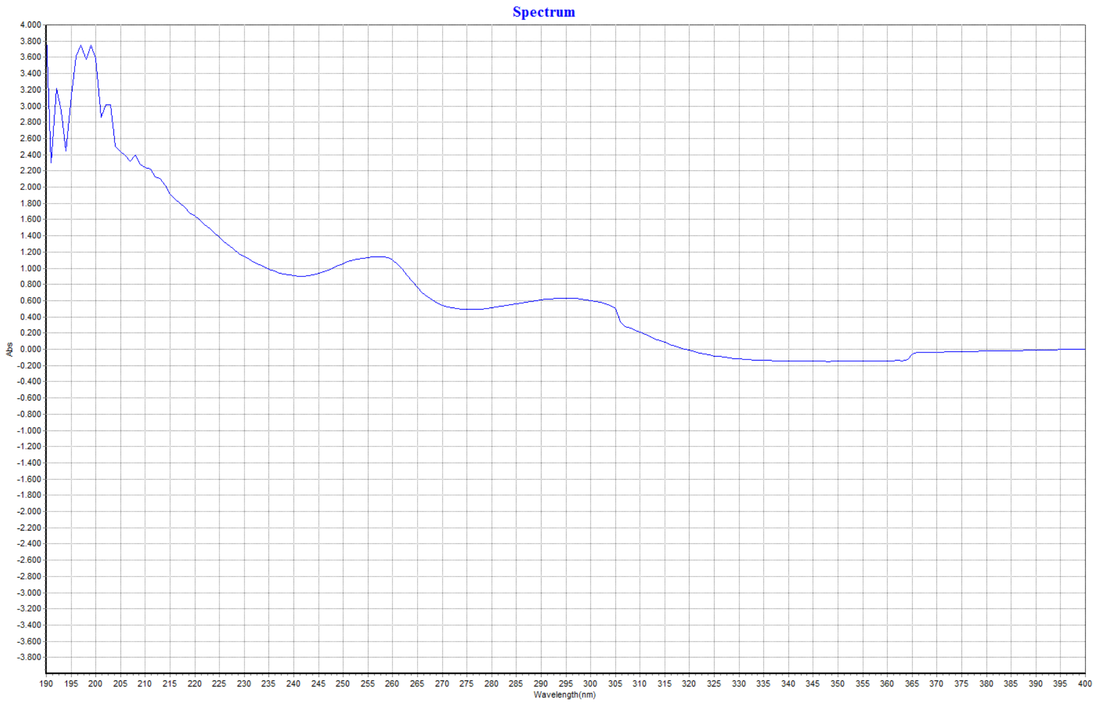

Figure S15. UV spectrum of compound 2

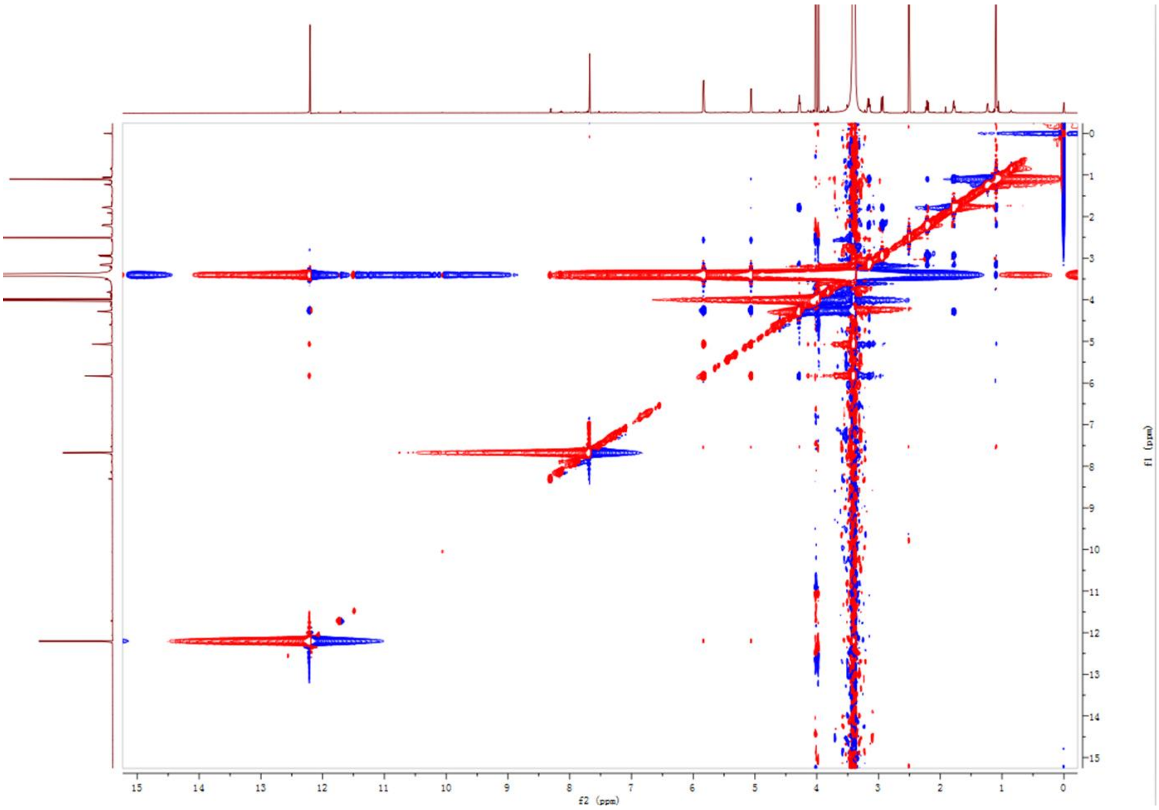

Figure S16. NOESY spectrum of compound 2

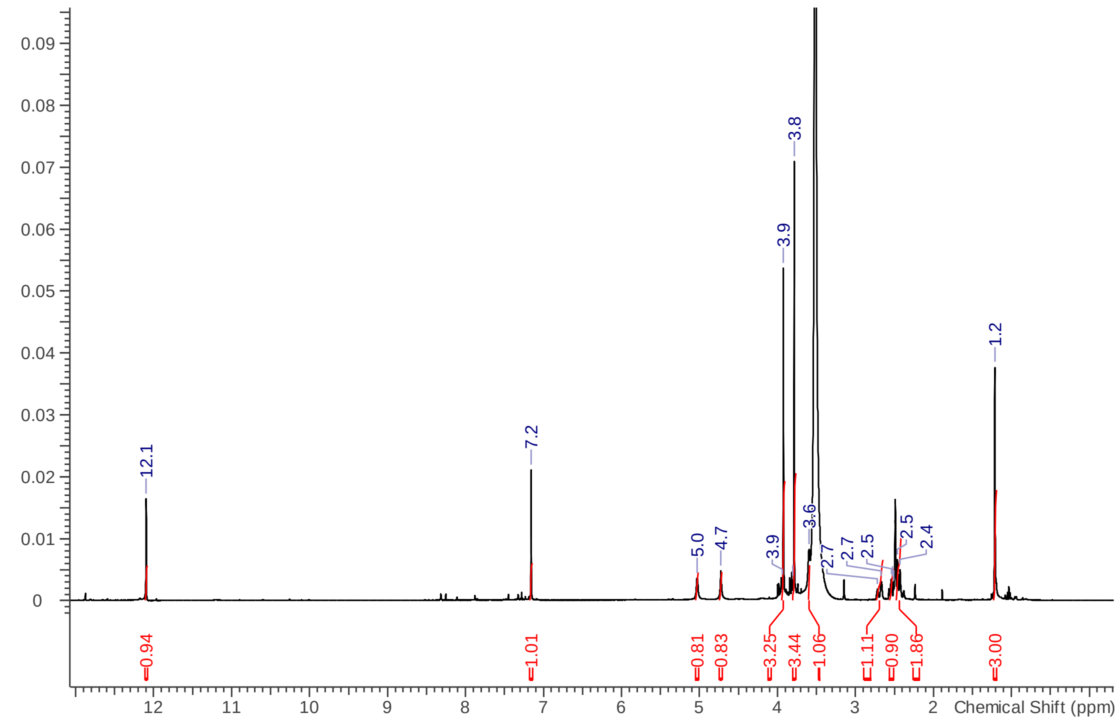

Figure S17. ^{1}H NMR spectrum of compound 3

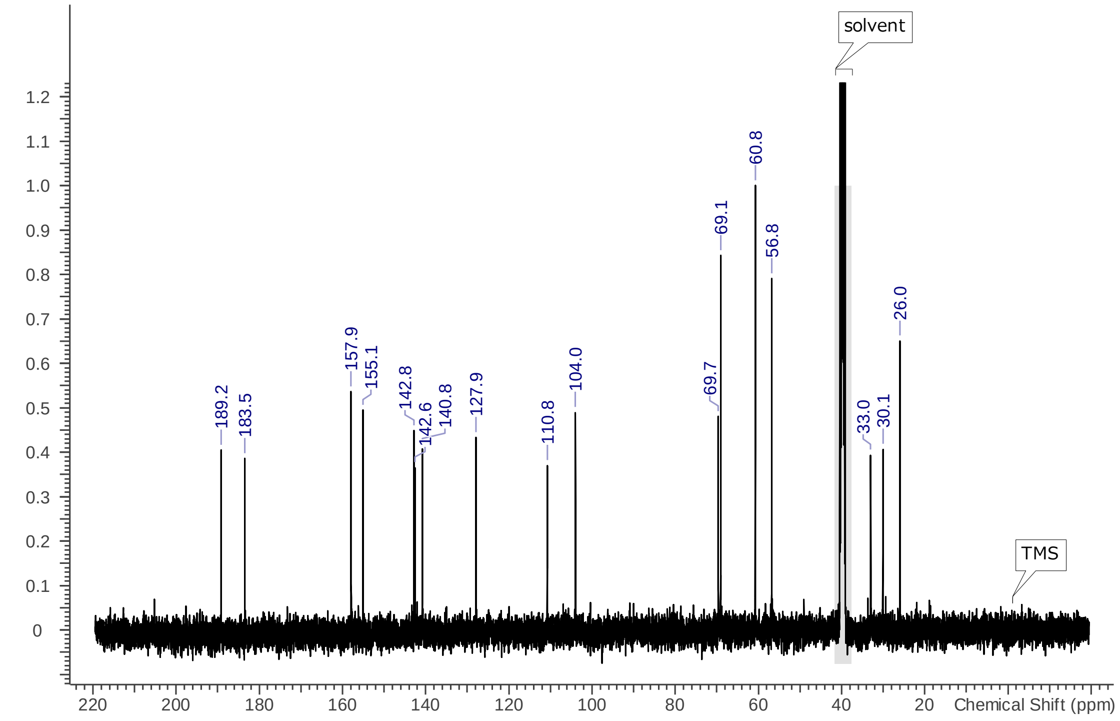

Figure S18. ^{13}C NMR spectrum of compound 3

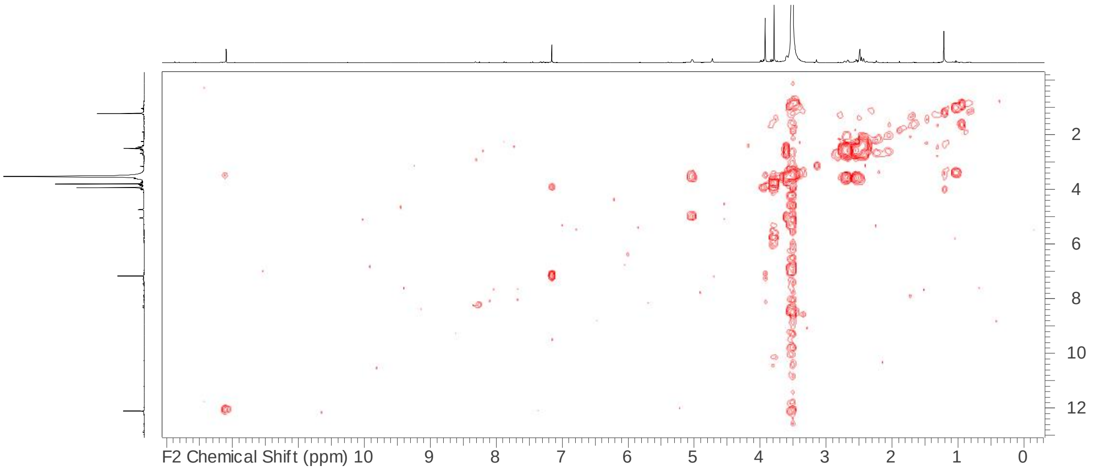

Figure S19. ^{1}H-^{1}H COSY spectrum of compound 3

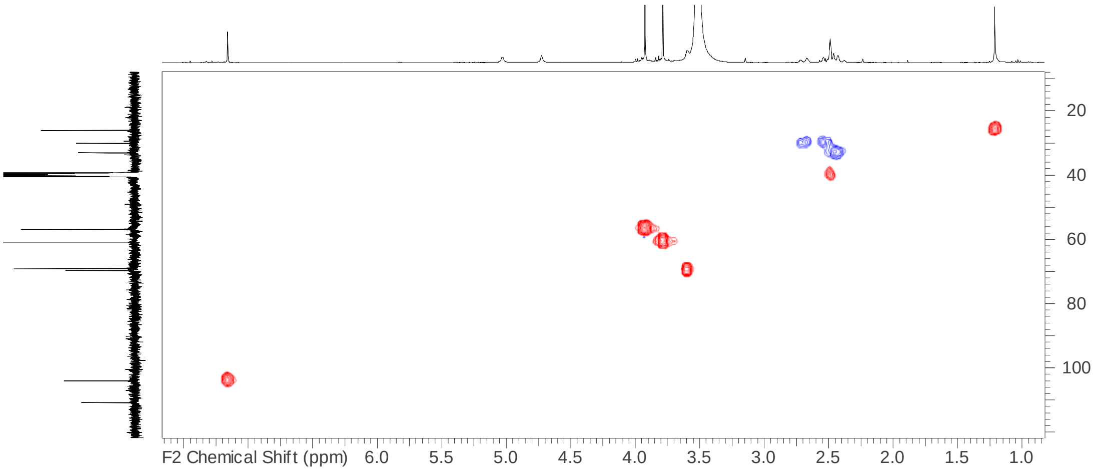

Figure S20. HSQC spectrum of compound 3

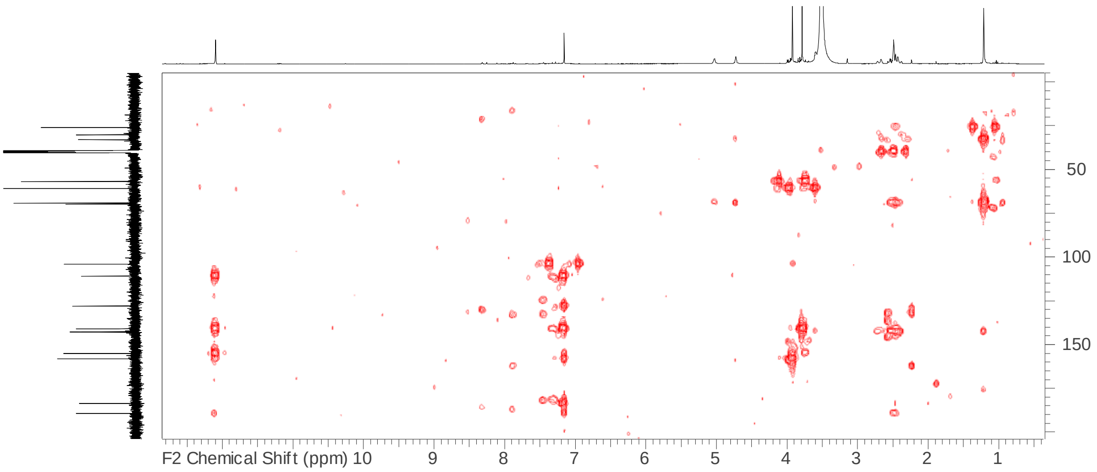

Figure S21. HMBC spectrum of compound 3

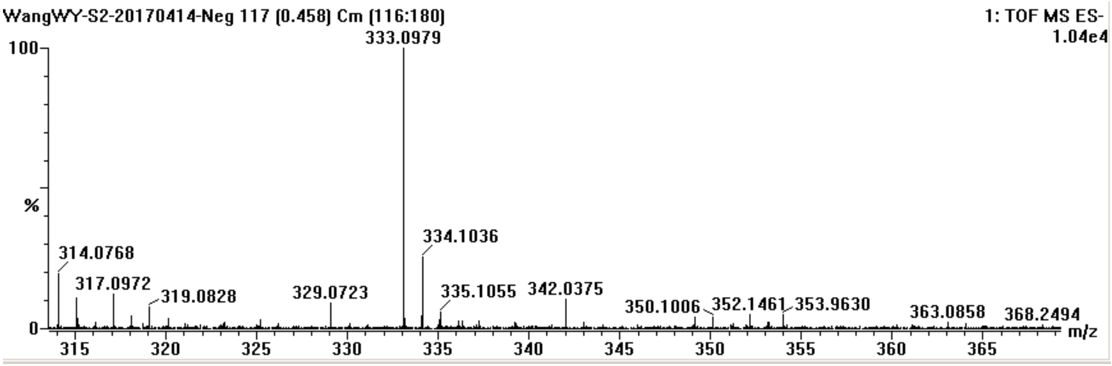

Figure S22. HRESIMS spectrum of compound 3

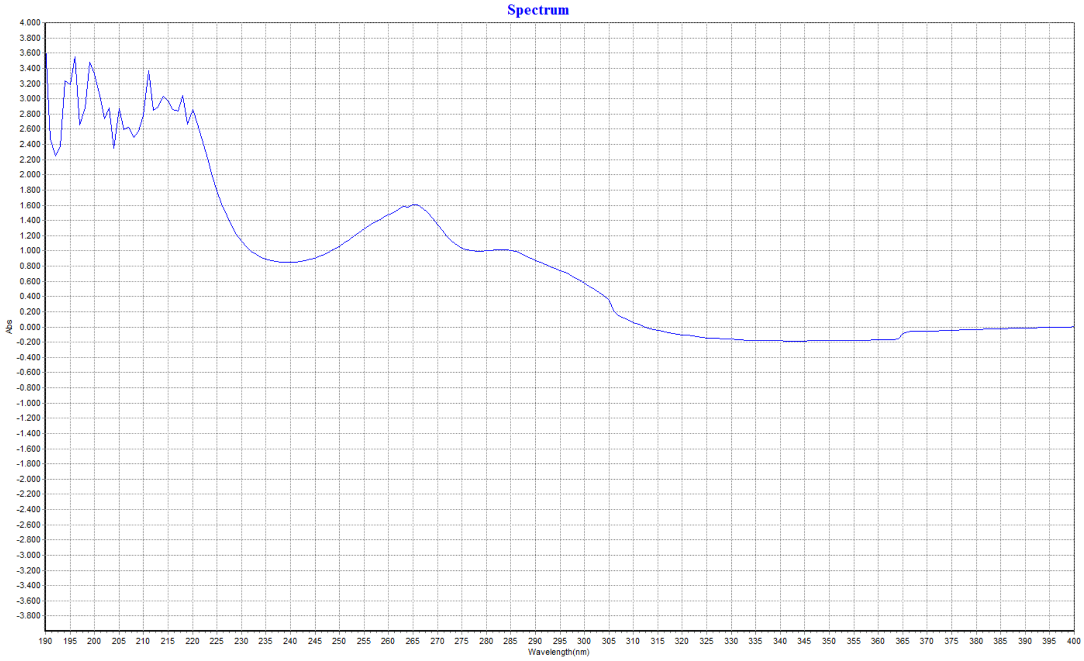

Figure S23. UV spectrum of compound 3

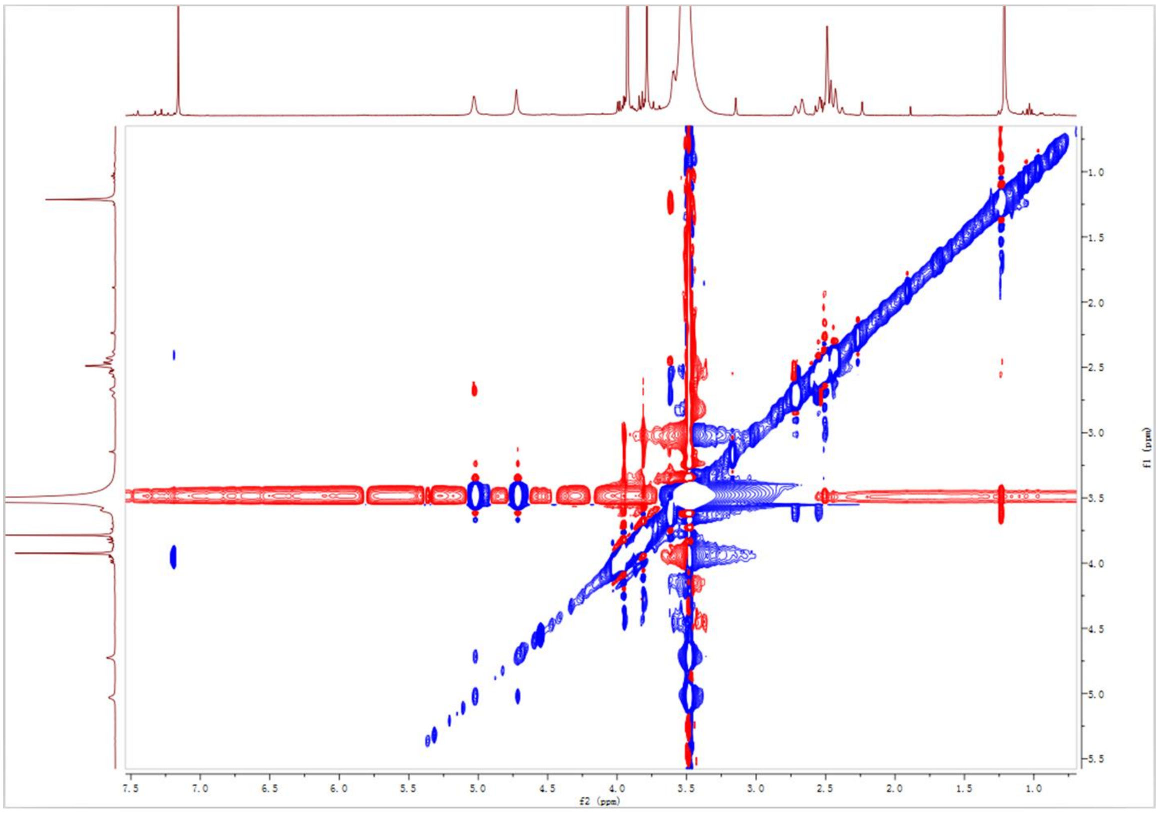

Figure S24. NOESY spectrum of compound 3

Table S1. Energies of the dominative conformers at MMFF94 force field of compound 2 & 3

Configuration	Conformer	Energy(kcal/mol)
1*S*, 2*S*, 3*R*-2	1	137.97
	2	149.21
	3	149.92
	4	156
	5	163.91
1*R*, 2*R*, 3*S*-2	1	141.2
	2	152.77
	3	154.9
	4	159.02
	5	166.91
2*R*, 3*S*-3	1	90.07
	2	90.69
	3	90.76
	4	91.24
2*S*, 3*R*-3	1	89.92
	2	90.08
	3	90.54
	4	90.70

marine drugs MDPI

Table S2. Energies of the conformers at B3LYP/6-311G** of compound 2 & 3 in methanol

Configuration	Conformation	Structure	E (Hartree)	E (kcal/mol)	Population (%)
1*S*, 2*S*, 3*R*-**2**	1	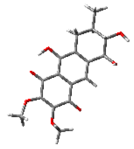	-1185.52017	-743925.1326	39.19
	2	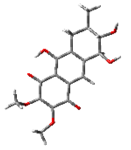	-1185.519535	-743924.7341	19.99
	3	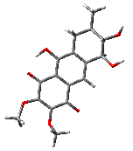	-1185.519373	-743924.6322	16.82
	4	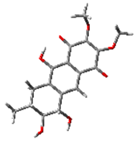	-1185.519576	-743924.7594	20.86

	5	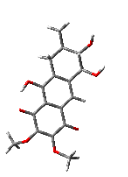	-1185.51779	-743923.6389	3.14
1*R*, 2*R*, 3*S*-**2**	1	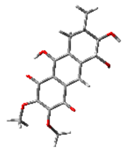	-1185.52017	-743925.1326	39.19
	2	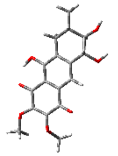	-1185.519535	-743924.7341	19.99
	3	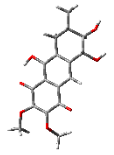	-1185.519373	-743924.6322	16.82
	4	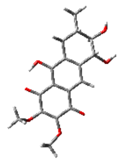	-1185.519576	-743924.7594	20.86

	5	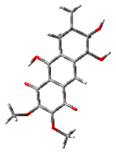	-1185.51779	-743923.6389	3.14
2*R*, 3*S*-**3**	1	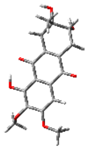	-1185.51779	-743923.6389	3.14
	2	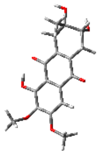	-1183.866035	-742887.1467	38.1
	3	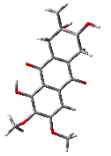	-1183.86602	-742887.1375	37.51
	4	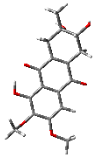	-1183.86501	-742886.5041	12.86

2*S*, 3*R*-**3**	1	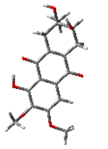	-1183.866032	-742887.1448	38.02
	2	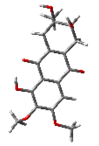	-1183.866022	-742887.1388	37.63
	3	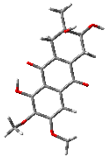	-1183.86501	-742886.5036	12.87
	4	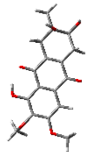	-1183.864903	-742886.4363	11.48

marine drugs

Text S1: ITS1-5.8S-ITS2 rDNA sequence of strain SP2-8-1

GenBank flat file:

```
LOCUS       MF716581                526 bp    DNA     linear   PLN
21-AUG-2017
DEFINITION  Aspergillus tritici strain sp2-8-1 18S ribosomal RNA gene, partial
            sequence; and internal transcribed spacer 1, 5.8S ribosomal RNA
            gene, and internal transcribed spacer 2, complete sequence.
ACCESSION   MF716581
VERSION     MF716581
KEYWORDS    .
SOURCE      Aspergillus tritici
  ORGANISM  Aspergillus tritici
            Eukaryota; Fungi; Dikarya; Ascomycota; Pezizomycotina;
            Eurotiomycetes; Eurotiomycetidae; Eurotiales; Aspergillaceae;
            Aspergillus.
REFERENCE   1  (bases 1 to 526)
  AUTHORS   Wang,W. and Liao,Y.
  TITLE     Chemical compostion of a coral-derived Aspergillus tritici strain
            sp2-8-1
  JOURNAL   Unpublished
REFERENCE   2  (bases 1 to 526)
  AUTHORS   Wang,W. and Liao,Y.
  TITLE     Direct Submission
  JOURNAL   Submitted (21-AUG-2017) Key Laboratory of Marine Biogenetic
            Resources, Third Institute of Oceanography, State Oceanic
            Administration, 178 Daxue Road, Xiamen, Fujian 361005, China
COMMENT     ##Assembly-Data-START##
            Sequencing Technology :: Sanger dideoxy sequencing
            ##Assembly-Data-END##
FEATURES             Location/Qualifiers
     source          1..526
                     /organism="Aspergillus tritici"
                     /mol_type="genomic DNA"
                     /strain="sp2-8-1"
                     /isolation_source="coral"
                     /db_xref="taxon:478866"
                     /country="Malaysia"
                     /identified_by="Yanyan Liao"
     misc_RNA        <1..526
                     /note="contains 18S ribosomal RNA, internal transcribed
                     spacer 1, 5.8S ribosomal RNA, and internal transcribed
                     spacer 2"
ORIGIN
        1 gaacctgcgg aaggatcatt accgagtgag ggtttctccg aagcccaacc tcccacccgt
       61 gtataccgta ccctgttgct tcggcgggcc cgcctcacgg ccgccggggg gcctcgcgcc
      121 cccgggcccg cgcccgccga agaccccaac acgaacactg tctgaaaagt gcagtctgag
      181 tcgattgtta ccaatcagtc aaaactttca acaatggatc tcttggttcc ggcatcgatg
      241 aagaacgcag cgaaatgcga taactaatgt gaattgcaga attcagtgaa tcatcgagtc
```

301 tttgaacgca cattgcgccc cctggtattc cggggggcat gcctgtccga gcgtcattgc
361 tgccctcaag cacggcttgt gtgttgggcc ccgtccccgg tacccccggg gacgggcccg
421 aaaggcagcg gcggcaccgc gtccggtcct cgagcgtatg gggctttgtc acccgctccg
481 caggcccggc cggcgccagc cgaccaaccc aaccattttc tacagg
//
